# The Innate Alarm System and Subliminal Threat Presentation in Posttraumatic Stress Disorder: Neuroimaging of the Midbrain and Cerebellum

**DOI:** 10.1177/2470547018821496

**Published:** 2019-02-05

**Authors:** Braeden A. Terpou, Maria Densmore, Janine Thome, Paul Frewen, Margaret C. McKinnon, Ruth A. Lanius

**Affiliations:** 1 Department of Neuroscience, Western University, London, Ontario, Canada; 2 Department of Psychiatry, Western University, London, Ontario, Canada; 3 Imaging Division, Lawson Health Research Institute, London, Ontario, Canada; 4 Department of Theoretical Neuroscience, Central Institute of Mental Health Mannheim, Medical Faculty Mannheim, Heidelberg University, Heidelberg, Germany; 5 Department of Psychology, Western University, London, Ontario, Canada; 6 Mood Disorders Program, St. Joseph’s Healthcare, Hamilton, Ontario, Canada; 7 Department of Psychiatry and Behavioural Neurosciences, McMaster University, Hamilton, Ontario, Canada; 8 Homewood Research Institute, Guelph, Ontario, Canada

**Keywords:** posttraumatic stress disorder, neuroimaging, midbrain, subliminal threat, trauma, periaqueductal gray, cerebellum

## Abstract

**Background:**

The innate alarm system, a network of interconnected midbrain, other brainstem, and thalamic structures, serves to rapidly detect stimuli in the environment prior to the onset of conscious awareness. This system is sensitive to threatening stimuli and has evolved to process these stimuli subliminally for hastened responding. Despite the conscious unawareness, the presentation of subliminal threat stimuli generates increased activation of limbic structures, including the amygdala and insula, as well as emotionally evaluative structures, including the cerebellum and orbitofrontal cortex. Posttraumatic stress disorder (PTSD) is associated with an increased startle response and decreased extinction learning to conditioned threat. The role of the innate alarm system in the clinical presentation of PTSD, however, remains poorly understood.

**Methods:**

Here, we compare midbrain, brainstem, and cerebellar activation in persons with PTSD (n = 26) and matched controls (n = 20) during subliminal threat presentation. Subjects were presented with masked trauma-related and neutral stimuli below conscious threshold. Contrasts of subliminal brain activation for the presentation of neutral stimuli were subtracted from trauma-related brain activation. Group differences in activation, as well as correlations between clinical scores and PTSD activation, were examined. Imaging data were preprocessed utilizing the spatially unbiased infratentorial template toolbox within SPM12.

**Results:**

Analyses revealed increased midbrain activation in PTSD as compared to controls in the superior colliculus, periaqueductal gray, and midbrain reticular formation during subliminal threat as compared to neutral stimulus presentation. Controls showed increased activation in the right cerebellar lobule V during subliminal threat presentation as compared to PTSD. Finally, a negative correlation emerged between PTSD patient scores on the Multiscale Dissociation Inventory for the Depersonalization/Derealization subscale and activation in the right lobule V of the cerebellum during the presentation of subliminal threat as compared to neutral stimuli.

**Conclusion:**

We interpret these findings as evidence of innate alarm system overactivation in PTSD and of the prominent role of the cerebellum in the undermodulation of emotion observed in PTSD.

## Introduction

The innate alarm system (IAS) is a network of brain structures serving the rapid detection of evolutionarily relevant and negatively valenced stimuli in the environment that can function during subliminal presentation.^1^ Subliminal stimuli defines information from the environment that is not perceived consciously. These stimuli are nonconscious since processing is predominantly restricted to a series of interconnected midbrain, brainstem, and thalamic nuclei that cannot support conscious processing due to reduced cortical engagement. These nuclei transmit sensory information that bypass primary cortices and directly innervate limbic and arousal brain circuitry.^1,2^ Through bypassing the cortex, the stimuli can be processed more rapidly thus conferring an evolutionary advantage when responding quickly to a threat in the environment.^3^ The IAS was identified via previous studies that presented fearful and neutral facial expressions to subjects very briefly such that they could not consciously discriminate between the expressions.^1^ Despite participants’ inability to discriminate these stimuli, subliminal fear presentation evoked an increase in brain activation at the level of the midbrain in the superior colliculus, lower brainstem in the locus coeruleus, and limbic circuitry in the amygdala.^1^ In addition to faces, the IAS response has been reported for the subliminal presentation of body posture cues, eye contact, and trauma-related words.^4,5^

Critically, posttraumatic stress disorder (PTSD) is associated with overactive threat detection circuitry as the result of trauma exposure.^4–6^ In PTSD, traumatic experiences promote attentional biases toward threat stimuli by way of elevated fear responses coupled with reduced extinction.^7^ Here, the threat bias in PTSD is evidenced by increased startle responses and emotional dysregulation of limbic circuitry during the presentation of consciously perceived fearful or trauma-related stimuli.^7–9^ PTSD is further associated with difficulties in extinguishing prior learned fear, as indicated by increased amygdala activation and skin conductance during extinction phases of learning, as compared to controls.^10,11^ Moreover, these neural and autonomic alterations are mirrored during the subliminal presentation of threat.^4,5,9,12^ Structures of the IAS showing increased activation in PTSD during the presentation of subliminal threat include the amygdala,^13,14^ parahippocampal gyrus,^15^ brainstem,^9,13^ and midbrain.^9,13^ Importantly, hyperactive amygdala activation is not a consistent finding for research that employs affect-related stimuli more generally in persons with PTSD as compared to controls.^4,15,16^ Here, hyperactive amygdala findings within the PTSD literature may be contingent upon the data analysis approach (i.e., whole-brain vs. region-of-interest (ROI)) as well as the comparison subjects employed (i.e., healthy controls vs. trauma-exposed controls).^17^ However, studies employing subliminal and supraliminal stimuli routinely elicit greater amygdala activation in PTSD as compared to control subjects.^18–20^ Taken together, these findings support the notion of a hyperactive IAS in PTSD toward threat.^6^ However, it remains unknown the contribution that specific low-level structures, contained within the midbrain, lower brainstem, and cerebellum, have toward the physiological signatures displayed in PTSD. Greater specificity of threat-detection circuitry could improve our clinical understanding of the disorder.

The physiological signatures that indicate a threat response are coordinated by low-level brain structures that alter the activation of opposing nervous systems. The autonomic nervous system (ANS) is the central system for responding to threat in the environment. The ANS is a division of nerve fibers that supply muscles and glands to regulate bodily functions without the need for conscious control. The sympathetic and parasympathetic branches of the ANS enact active (i.e., fight, flight) and passive (i.e., faint, tonic immobility) defensive responses, respectively.^21,22^ These responses are characterized by dissociable changes in physiology, with active and passive defenses exemplifying sympathoexictation (i.e., hypertension, tachycardia) and sympathoinhibition patterns (i.e., hypotension, bradycardia), respectively.^23^

The periaqueductal gray is a midbrain structure that coordinates the defensive responses via activation of its opposing subunits—the dorsolateral periaqueductal gray (active defenses) and ventrolateral periaqueductal gray (passive defenses).^24,25^ The periaqueductal gray is heavily connected with the IAS, as it receives projections from limbic and subcortical structures which evaluate the emotional valence of stimuli.^26^ Moreover, the periaqueductal gray shares connectivity with the insula, a cortical region involved in the regulation of the ANS.^20,27^ Critically, both the periaqueductal gray and insula show increased activation in PTSD during symptom provocation.^9,28^ In addition, the periaqueductal gray exhibits increased resting-state functional connectivity with areas underlying emotional reactivity in PTSD as compared to controls.^29^ These reports converge with a study by Felmingham and colleagues^9^ where persons with PTSD displayed increased periaqueductal gray activation as compared to controls during subliminal threat presentation.^9^ Taken together, these findings suggest that overactive threat detection circuitry may promote periaqueductal gray-mediated physiological changes which can present as symptoms of hypervigilance, or, in severe cases, defensive responses in PTSD (i.e., fight or flight, tonic immobility) pending on the level of threat perceived.

The midbrain reticular formation is another midbrain area associated with threat stimuli. The midbrain reticular formation is a combination of nuclei that occupy a large portion of the midbrain tegmentum.^30^ The initial functional characterization of the midbrain reticular formation associated it with transitions in brain states, for example, transitioning from a sleeping to waking state.^31^ These transitions are guided by ascending and descending cholinergic projections from the midbrain reticular formation throughout the ascending reticular activating system (ARAS).^32,33^ The ARAS refers to a network of connected brainstem, midbrain, and thalamic nuclei that drive cholinergic and glutamatergic projections to the cortex.^34,35^ These projections assist in the generation and maintenance of arousal states reflected in the limbic and prefrontal cortices.^30,36,37^ Moreover, the midbrain reticular formation receives crudely processed sensory information from the superior colliculus—the central structure of the IAS.^1,38^ In concert with the superior colliculus, the midbrain reticular formation can produce involuntary changes in gaze direction when stimulated in primates.^39,40^ Together, the evidence suggests that cholinergic projections from the midbrain reticular formation engage arousal and limbic circuitry following the detection of a threat.^38,39,41–43^ Moreover, this system appears capable of initiating strong aversive emotional states during threat display in rats.^44–46^ Despite the known role of the midbrain reticular formation in the generation of arousal states, to our knowledge, it remains unclear how this region contributes to symptom expression in PTSD.

The cerebellum is a hindbrain region involved in the regulation of emotional states that may function in concert with the IAS.^47,48^ The cerebellum shares connectivity with midbrain and limbic circuitry and elicits activation in the presence of threat.^49,50^ Moreover, stimulation of the cerebellum can induce activation in mesolimbic circuitry, and cerebellar lesions are associated with symptoms of emotion dysregulation.^51–53^ The right cerebellar lobule V is a cerebellar region with a preference for aversive stimuli, as indicated by increased activation to fearful as compared to neutral facial expressions in healthy participants.^49,54^ The pattern of activation in the right cerebellar lobule V mirrors that of the amygdala, lending support to their coinvolvement during evoked aversive states.^54,55^ Whereas amygdala activation maintains an aversive state, cerebellar activation may attenuate the emotional response.^48,56^ The latter finding is supported by studies employing slow repetitive transcranial magnetic stimulation to inhibit cerebellar function during emotion generation.^48^ During inhibition of the cerebellum, participants report heightened aversive states and greater amygdala activation.^48^ In PTSD, the right cerebellar lobule V demonstrates a resting-state decoupling with multisensory cortices including the temporoparietal junction and parietal operculum.^57^ Moreover, PTSD is associated with a general decrease in right cerebellar activation during emotion provocation.^58^ To the extent that the cerebellum regulates aversive states, reductions in its function may promote IAS overactivation in PTSD.

The IAS is a network of low-level structures that process subliminal stimuli and may demonstrate altered activation in PTSD.^1,6^ The contribution of specific midbrain, brainstem, and cerebellar structures to the exaggerated threat response observed in PTSD is not well understood. Accordingly, our aim was to investigate neural activation in PTSD during subliminal threat presentation using improved normalization of the functional magnetic resonance imaging (fMRI) data generated from these low-level structures. We hypothesized that individuals with PTSD would show increased activation during the presentation of subliminal threat stimuli within the midbrain by way of the overrecruitment of the IAS.^6^ For nontrauma-exposed controls, we hypothesized that they would demonstrate increased right cerebellar activation as compared to PTSD as a reflection of their enhanced capacity to regulate affect.^48,54^

## Methods

### Participants

This study was approved by the Health Sciences Research Ethics Board of Western University and adhered to the standards set forth by the Tri-Council Policy. In total, 46 English-fluent participants were recruited for the study, 26 met the criteria for a primary diagnosis of PTSD and 20 were included as healthy, nontrauma-exposed controls. Participants were recruited by the London Health Services Centre via referrals from physicians, community clinics, mental health professionals, and advertisements in the community. Data generated on this sample and paradigm have been analyzed separately and reported in previous work.^5,59,60^ All participants provided written and informed consent for their involvement.

Exclusion criteria for the study included incompatibility with scanning requirements, previous neurologic and developmental illness, pregnancy, comorbid schizophrenia or bipolar disorder, alcohol or drug abuse within six months prior to scanning, or a history of head trauma. Diagnoses for PTSD were ascertained using the Clinician Administered PTSD Scale (CAPS) (CAPS-IV cutoff score >50 for PTSD diagnosis) as well as a Structured Clinical Interview for DSM-IV Axis I disorders.^61,62^ In terms of the type of trauma experienced, 23 of the 26 persons with PTSD experienced childhood interpersonal trauma while the remaining 3 of the 26 persons experienced a personal threat of life or witnessed a violent death. Control subjects did not meet any current or lifetime criteria for psychiatric disorders. In addition, the Childhood Trauma Questionnaire (CTQ),^63^ Multiscale Dissociative Inventory (MDI),^64^ and Beck’s Depression Inventory were administered.^65^ Following scanning, participants completed the State-Trait Anxiety Inventory (STAI)^66^ and the Responses to Script Driven Imagery (RSDI)^67^ questionnaire to assess any perceptible fluctuations in state and trait anxiety, and PTSD symptoms related to the paradigm. Finally, the Clinician Administered Dissociative States Scale (CADSS)^68^ was administered to determine whether persons experienced a dissociative episode during fMRI scanning.

### Experimental Task

The fMRI procedure and psychophysical thresholds were based on published methods for the presentation of subliminal and supraliminal stimuli.^9,59,69^ All stimuli had a subliminal and supraliminal presentation over two consecutive sessions which were counterbalanced across subjects and involved a 2-minute rest period between sessions. Cues represented both threat-related (fearful facial expressions and personalized trauma words (TWs)) and neutral (neutral facial expressions and words) stimuli presented in a pseudo-randomized block design. Word cues were subject-specific, with TWs generated with respect to a patient’s individualized trauma experience or, in the case of controls, an aversive experience. Neutral words (NWs) were selected if they had not elicited a strong positive or negative reaction during prescanning exposure to the word. All words were matched for syllable and letter length. Each block (NWs, TWs, neutral faces (NFs), fearful faces (FFs)) was repeated five times in a fixed order to the participant. Face stimuli were three-dimensional and selected from a standardized database.^70^ Each block consisted of eight repetitions of the stimulus as either subliminal or supraliminal. Subliminal stimuli were presented for 16 ms and separated by a jittered interstimulus interval that varied in duration from 823 to 1823 ms. Subliminal presentation of stimuli was masked (mask duration: 161 ms) to ensure preconscious processing.^1^ Supraliminal stimuli were presented for 500 ms and separated by a jittered interstimulus interval of 500 to 1500 ms. A button press task was implemented between stimulus presentation blocks to ensure sustained attention throughout the scanning session (letter recognition; 4500 ms). Finally, each run was preceded by a 30-s rest period which was used as an implicit baseline for comparisons in subsequent analyses (stimuli: fixation cross).

### fMRI Data Acquisition

Functional images were collected on a 3.0 T whole-body MRI scanner (Siemens Biograph mMR, Siemens Medical Solutions, Erlangen, Germany) using a 32-channel phased array head coil. T1-weighted anatomical images were collected with 1-mm isotropic resolution (MP-RAGE, time resolution (TR)/echo time (TE)/time interval (TI) = 2300 ms/2.98 ms/900 ms, flip angle (FA) 9°, field of view (FOV) = 256 mm × 240 mm × 192 mm, acceleration factor = 4, total acquisition time = 192 s). Sixty-four whole-brain, 2-mm-thick imaging planes for blood-oxygen-level dependent (BOLD) fMRI were generated parallel to the anterior commissure – posterior commissure (AC–PC) line. Functional data were acquired using the manufacturer’s standard gradient-echo EPI pulse sequence (single shot, blipped EPI) with interleaved slice acquisition order and tridimensional perspective correction and an isotropic resolution of 2 mm ((FOV = 192 mm × 192 mm × 128 mm (94 × 94 matrix, 64 slices), TR/TE = 3000 ms/20 ms, FA = 90° (FOV, TR, TE, and FA)).

### fMRI Analysis Using Spatially Unbiased Infratentorial Template Toolbox

To improve the normalization procedure and receive a clearer depiction of midbrain, brainstem, and cerebellar activation, data were normalized to the spatially unbiased infratentorial template (SUIT).^71,72^ The SUIT toolbox offers a high-resolution atlas template of the cerebellum and brainstem with improved voxel-by-voxel normalization of fMRI. The SUIT toolbox functions on Statistical Parametric Mapping (SPM12, Wellcome Trust Centre for Neuroimaging, London, UK: http://www.fil.ion.ucl.ac.uk/spm) within MATLAB 9.2 (R2017a, Mathworks Inc., MA) and contains several preprocessing steps. First, anatomical images were reoriented in statistical parametric mapping (SPM) where the horizontal plane was defined approximately according to the AC–PC line. Second, functional images were reoriented to correspond to the reoriented anatomical image. Third, subject-specific functional volumes were realigned to the first volume of each session to correct for movement in the scanner and then resliced to a voxel size of 2 × 2 × 2 mm^3^. At this time, six realignment parameters for changes in motion across the different planes and an artifact detection tools (ART) regressor for global movement correction were saved. Fourth, subject-specific brainstem and cerebellum were isolated and cropped from the T1-weighted anatomical images in order to focus on the infratentorial structures of interest. Fifth, individual cropped anatomical images of the brainstem and cerebellum were normalized into the SUIT atlas template. During this step, a subject-specific transformation matrix was generated for the linear part of the normalization that deforms each cerebellum to provide optimal correspondence to the SUIT template.^73^ Sixth, functional volumes were resliced into SUIT space in order to align functional images with the SUIT-normalized anatomical images by applying the subject-specific transformation matrix. Finally, a three-dimensional isotropic 4-mm full-width at half-maximum Gaussian kernel was applied to each set of SUIT-resliced functional data to smooth the data in accordance with previous methods using SUIT preprocessing.^74,75^

### Statistical Analysis

#### Within-Subject Analysis

In the first-level analyses, a fixed-effects model was generated in which the time series of eight conditions (subliminal: TW, NW, FF, and NF and supraliminal: TW, NW, FF, and NF) were convolved to the default canonical hemodynamic response function. The button task and realignment parameters were included as regressors of no interest. An ART regressor, which accounts for effects of movement and global signal correction (version 2015-10; Gabrieli Lab, McGovern Institute for Brain Research, Cambridge, MA), was added as a within-subject covariate of no interest as well. Software default thresholds for ART regressor outliers were selected (global signal threshold = 9.0 mm, absolute subject motion threshold = 2.0 mm, rotational threshold = .05 mm, scan-to-scan subject motion = 2.0 mm, and scan-to-scan subject rotation = 0.02 mm). At this time, contrast images were created for the subliminal presentation of trauma-related words minus the subliminal presentation of NWs (subliminal: TW > NW) as well as the subliminal presentation of FFs minus the subliminal presentation of NFs (subliminal: FF > NF) for each subject. As well, contrasts for the supraliminal presentation of trauma minus NWs (supraliminal TW > NW) and fearful minus NFs (supraliminal: FF > NF) were also conducted. These contrasts were carried forward to the second level for random-effects group comparisons.

#### Group Analyses

In the second-level analyses, a full-factorial analysis of variance was conducted on the data to examine the 2 × 2 × 2 interaction between group (PTSD, controls), conscious level (subliminal, supraliminal), and stimulus contrast condition (TW > NW, FF > NF). These comparisons were analyzed using random-field theory as implemented by SPM12. Variances were set to unequal to account for differences in group sizes. While exploring random-effects group comparisons across SUIT space, an initial significance threshold was set to *p*-uncorrected < .005, *k* ≥ 5. An initial liberal threshold was employed due to the analyses being novel and to allow for the overall trends of the data to be observed using a less-conservative threshold.

Subsequent ROI analyses were conducted to restrict the voxels of examination to regions involved in the IAS and associated with PTSD. No subject-specific coordinates were employed. All results for the ROI analyses were thresholded at *p-*family wise error (FWE) < .05, *k* ≥ 5. Identification of brain regions were obtained by using the cerebellar probabilistic atlas template for SUIT as well as the ascending arousal network (AAN) atlas which details the position of many brainstem nuclei in MNI space.^34,71^ The ROI used for the analyses was a single mask generated by combining midbrain and cerebellar structures. From the midbrain, the bilateral superior colliculus, periaqueductal gray, and midbrain reticular formation were selected. Masks for the periaqueductal gray and midbrain reticular formation were adopted from the AAN atlas due to its strong structural and functional validation and free access.^34^ The superior colliculus mask was generated using PickAtlas software (WFU Pickatlas, version 2.5.2)^76^ and followed the anatomical description provided by Martin.^77^ From the cerebellum, the particular coordinates for the right cerebellar lobule V were adopted from the SUIT template.^71^ Finally, the four regions were merged into a single mask using the imcalc toolbox provided in SPM12 (http://tools.robjellisnet) and verified using MRIcron.^78^

#### Clinical Correlations

A multiple regression was conducted within the PTSD group to determine whether clinical scores correlated with brain activation within the conditions of interest. In this case, we were interested in the contrasts of the subliminal presentation of TW > NW and FF > NF. Activation within the PTSD group was correlated with symptom scores of reexperiencing (CAPS criterion B), avoidance (CAPS criterion C), negative alterations in cognition and mood (CAPS criterion D), and dissociation (MDI Depersonalization and Derealization subscales). For the CAPS scores, each criterion was analyzed separately as well as the sum of frequency and intensity scores for B, C, and D.^61^ Moreover, correlations of PTSD activation were conducted with trauma history (CTQ) and state symptom scores (STAI, RSDI, and CADSS). The analysis was thresholded initially at *p-*uncorrected < .005 with follow-up ROIs using *p*-FWE < .05, *k* ≥ 5.

## Results

### Demographics and Clinical Measures

Independent sample *t* tests did not reveal any significant differences between PTSD and the control group with respect to demographic measures. As predicted, persons with PTSD scored significantly higher on total scores for the CAPS, MDI, and CTQ (see Table 1).

**Table 1.** Clinical and Demographic Information.

Measure	PTSD (N = 26) M ± SD	Healthy controls (N = 20) M ± SD	χ^2^ *p*	T test *p*
Years of age	38.8 ± 12.2	32.5 ± 11.6	.088	–
Sex (n)	Male = 11, female = 15	Male = 10, female = 10	.604	–
Employment status (n)	Employed = 18, unemployed = 7	Employed = 17, unemployed = 3	.297	–
CAPS total	70.6 ± 11.9	0.94 ± 2.9	–	<.001
CTQ: Emotional abuse	14.5 ± 6.1	6.8 ± 3.1	–	<.001
Moderate cutoff met (n)	5	–	–	–
Severe cutoff met (n)	11	–	–	–
CTQ: Physical abuse	10.1 ± 6.4	5.7 ± 1.6	–	.004
Moderate cutoff met (n)	0	–	–	–
Severe cutoff met (n)	9	–	–	–
CTQ: Sexual abuse	13.4 ± 7.8	5.3 ± 1.1	–	<.001
Moderate cutoff met (n)	1	–	–	–
Severe cutoff met (n)	14	–	–	–
CTQ: Emotional neglect	13.5 ± 5.9	8.8 ± 4.2	–	.004
Moderate cutoff met (n)	2	–	–	–
Severe cutoff met (n)	10	–	–	–
CTQ: Physical neglect	10.2 ± 4.7	6.8 ± 2.7	–	.006
Moderate cutoff met (n)	5	–	–	–
Severe cutoff met (n)	6	–	–	–
MDI total	58.8 ± 21.6	33.7 ± 3.8	–	<.001
MDI depersonalization	7.8 ± 4.1	–	–	–
MDI derealization	9.5 ± 4.5	–	–	–
MDI depersonalization/ derealization	8.7 ± 4.1	–	–	–
Axis I comorbidities (current [past]) frequency	Major depressive disorder (8[9])			
	Dysthymic disorder (0[3])			
	PD w/o agoraphobia (0[1])			
	PD w/o agoraphobia (1[1])			
	Agoraphobia w/o PD (3)			
	Social phobia (4)			
	Specific phobia (2)			
	OCD (1[1])			
	Eating disorders (1[1])			
	Somatoform disorder (6)			
	Lifetime alcohol abuse or dependence [16]			
	Lifetime substance abuse or dependence [7]			

CAPS: Clinician Administered PTSD Scale; CTQ: Childhood Trauma Questionnaire; MDI: Multiscale Dissociation Inventory; OCD: obsessive-compulsive disorder; PD: panic disorder; PTSD: posttraumatic stress disorder; SD: standard deviation.

### Imaging Results

#### Within-Group Comparisons

No significant differences in neural activation were revealed for within-group, between-group, or clinical correlations for the contrast condition of the subliminal presentation of FF > NF as well as any supraliminal presentation contrasts. As a result, the results and discussion will focus specifically on the subliminal presentation of TW > NW.

All results were restricted to the SUIT space offered by the toolbox. For controls, no significant voxels were detected at the significance of *p*-FWE < .05, *k* ≥ 5. For the PTSD group, a significant cluster emerged with a peak-coordinate centered on the periaqueductal gray ((*x*: 0, *y*: −32, *z*: −11), *k* = 53, *p-*FWE = .013) during subliminal trauma-related words as compared to neutral stimulus presentation (see Table 2). This cluster also covered areas of the superior colliculus and midbrain reticular formation.

**Table 2.** Within-Group Differences in Spatially Unbiased Infratentorial Template Space.

Contrast	LR	Region	*k*	*p* (FWE-cor)	*z*	MNI coordinates		
						x	y	z
Subliminal TW > NW								
Control		None						
PTSD		Periaqueductal gray	53	.013	4.39	0	−32	−11

FWE: family wise error; LR: left or right (hemipshere); MNI: Montreal Neurological Institute; NW: neutral words; PTSD: posttraumatic stress disorder; TW: trauma word.

#### Between-Group Comparisons

**Figure 1.**
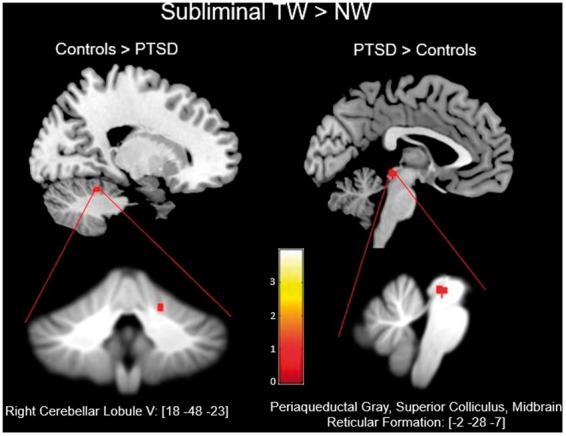
Details the exported clusters that reached significance for the contrasts of controls > PTSD and PTSD > controls during the subliminal presentation of TWs as compared to NWs. Below are the clusters as they appear on the SUIT template. NW: neutral words; PTSD: posttraumatic stress disorder; TW: trauma word.

Applying the ROI mask of the bilateral superior colliculus, periaqueductal gray, midbrain reticular formation, and right cerebellar lobule V to the partial-brain space yielded significant between-group results at *p*-FWE < .05, *k* ≥ 5. For the subliminal presentation of the contrast condition of TW > NW, control subjects demonstrated significantly greater activation as compared to the PTSD group at a peak-coordinate centered on the right cerebellar lobule V ((*x*: 18, *y*: −48, *z*: −23), *k* = 5, *p-*FWE = .019) (see Table 3). Conversely, the same contrast yielded greater activation in the PTSD group at a peak-coordinate centered on the periaqueductal gray, midbrain reticular formation, and superior colliculus ((*x*: −2, *y*: −28, *z*: −7), *k* = 13, *p-*FWE = .019) (Figure 1).

**Table 3.** Between-Group Differences in Spatially Unbiased Infratentorial Template Space for ROI Analysis.

Contrast	LR	Region	*k*	*p* (FWE-cor)	*z*	MNI coordinates		
						x	y	z
Subliminal TW > NW								
Control > PTSD	R	Cerebellar lobule V	5	.019	3.87	18	−48	−23
PTSD > Control		Periaqueductal gray/midbrain reticular formation/superior colliculus	13	.019	3.87	−2	−28	−7

FWE: family wise error; LR: left or right (hemipshere); MNI: Montreal Neurological Institute; NW: neutral words; PTSD: posttraumatic stress disorder; TW: trauma word.

#### Clinical Correlations

The whole SUIT brain analysis did not reveal any significant correlations between clinical scores and BOLD activation in the PTSD group during the multiple regression analysis. The follow-up ROI analysis yielded significant results at *p*-FWE < .05, *k* ≥ 5 for the subliminal contrast of TW > NW. The significant correlation was negative and emerged between scores on the MDI Depersonalization/Derealization subscales and BOLD activation in the right cerebellar lobule V ((*x*: 12, *y*: −56, *z*: −23), *k* = 11, *p*-FWE = .032) in the PTSD group (see Table 4).

**Table 4.** Correlations of Clinical Scores With BOLD Activation in Posttraumatic Stress Disorder Group for ROI Analysis.

Clinical measure (direction of effect)	LR	Region	*k*	*p* (FWE-cor)	*z*	MNI coordinates		
						x	y	z
Subliminal TW > NW								
MDI depersonalization/ derealization (negative)	R	Cerebellar lobule V	11	.032	3.77	12	−56	−23

BOLD: Blood-Oxygen-Level Dependent; FWE: family wise error; LR: left or right (hemipshere); MNI: Montreal Neurological Institute; MDI: Multiscale Dissociation Inventory; NW: neutral words; ROI: Region-of-Interest; TW: trauma word.

## Discussion

### Overview

To date, the PTSD neuroimaging literature has focused predominantly on the divergence of cortical networks in the pathological brain when compared to healthy controls. Here, theories have emerged that attempt to explain how PTSD symptoms arise as a result of dysfunction in top-down cortical networks. These theories, however, often neglect to incorporate midbrain, brainstem, and cerebellar involvement despite the reliance of the cortex on these structures. In the present study, we implemented a more precise analysis protocol with improved normalization of the midbrain, brainstem, and cerebellum to image persons with PTSD and healthy controls during the presentation of a subliminal threat as compared to a neutral stimulus. As predicted, midbrain regions associated with the IAS showed increases in activation during the viewing of trauma-related words in persons with PTSD as compared to controls. In controls Blood-Oxygen-Level Dependent, elevated activation in the subliminal threat condition was detected in the right cerebellar lobule V as compared to PTSD. Moreover, the right cerebellar lobule V was found to correlate negatively with MDI symptom scores of depersonalization/derealization in persons with PTSD. These different neural responses to subliminal threat provide novel evidence toward the alterations of low-level structures in PTSD, which, when considered together, may contribute to a more integrated understanding of this disorder.

### Between-Group Comparisons

Our analyses revealed increased response of the superior colliculus, periaqueductal gray, and midbrain reticular formation for the ROI analysis of the subliminal presentation of trauma-related words in PTSD as compared to controls. These results converge with studies involving participants with PTSD that revealed increased activation to threat when presented at,^28,79,80^ or below conscious threshold.^4,9,14,81^ In particular, our findings resemble those of Felmingham and colleagues^9^ who reported increased activation in the superior colliculus and periaqueductal gray of women with PTSD as compared to a control group during the presentation of a subliminal threat. We argue that these results provide evidence for the overactivation of threat-detection circuitries toward a pathological extreme in PTSD.^6^

The superior colliculus refers to a set of paired midbrain nuclei that are central to the function of the IAS. This structure transmits crude visual information to the pulvinar nuclei of the thalamus to form an alternative visual pathway that supports the act of saccadic eye movements.^1^ Moreover, this pathway is proposed to assist in detecting novel and evolutionarily relevant stimuli for rapid processing.^82,83^ Stimulation of the superior colliculus in rodents and in nonhuman primates can elicit approach or defensive responses in the form of orienting/pursuit eye movements (approach) or fight/flight responses (defense), respectively.^84,85^ These responses are subserved by distinct output projections from the superior colliculus.^86^ Interestingly, stimulation of the deep layers of the superior colliculus and the periaqueductal gray evoke a similar response of anxiety-like behaviors in rats such as freezing or flight.^87^ Alternatively, stimulation at a more rostral location of the superior colliculus elicits a response of orienting and approach in rats, similar to the pattern of response observed through stimulation of the midbrain reticular formation.^88,89^ Taken together, these complementary findings provide evidence of the co-engagement of the superior colliculus with the periaqueductal gray and midbrain reticular formation for the generation of defensive and orienting responses, respectively.

We interpret the increase in midbrain activation in the PTSD group observed in the present study as reflecting an overactivation of the IAS toward subliminal threat. Here, the superior colliculus may initiate a response following detection of the trauma-related words and transmit relevant information to the nearby midbrain.^1^ In turn, cholinergic projections may be sent from the midbrain reticular formation throughout the ARAS toward limbic and prefrontal cortices to engage arousal circuitry to better orient to the threat present.^32,38,41^ Simultaneously, information relayed to the periaqueductal gray may prompt a defensive cascade where subunits project to brainstem nuclei to initiate physiological changes communicated through the ANS.^22,24,27^ Here, the periaqueductal gray would coordinate the appropriate defensive response by evaluating certain characteristics of the threat as well as the situation in which it occurs. In addition, individual differences in trauma experience also effect the proclivity by which one defensive response is favored over another.^24^ In summary, this interpretation centered on the midbrain can account for many experimental characteristics of PTSD, including increased startle responses to threat,^7,9,90^ neutral cues,^91^ blunted,^92–94^ or exaggerated autonomic reactivity,^95,96^ as well as the inability to achieve a restful state.^29,57,97,98^

In addition, our analyses revealed significantly greater cerebellar activation in controls as compared to PTSD during the presentation of a subliminal threat. In particular, the increased response was generated in the right cerebellar lobule V, a lobule involved in the expression and regulation of aversive states.^54,55^ Within individuals with PTSD, activation of the right lobule V was found to correlate negatively with scores on the MDI for the Depersonalization/Derealization subscales. As dissociative symptom scores increased in the PTSD group, the activation of the right lobule V decreased. This finding converges with a resting-state study that showed reduced functional connectivity of the anterior cerebellum with cortical regions involved in multisensory integration and bodily self-consciousness in persons with PTSD who met the criteria for the dissociative subtype as compared to controls.^57^ Whereas we show that during threat display, persons with greater dissociative scores—and, hence, higher detachment from their emotions—have the lowest engagement of the right cerebellum, Rabellino and colleagues^57^ reveal that the dissociative subtype demonstrates reduced connectivity of the cerebellum, a region involved in emotion processing, with cortical areas that may ground emotions within the body. Furthermore, our results corroborate earlier studies that revealed a positive association between hyperarousal symptoms in PTSD and regional cerebral blood flow to the right lobule V.^99^ Whereas hyperarousal symptoms characterize a state of emotional undermodulation, dissociative symptoms reflect a state of emotional overmodulation.^94^ To the extent that the right cerebellum acts to regulate emotions, one would expect to observe opposing patterns of associated neural activation with dissociative and hyperarousal symptom measures. Taken together, studies distinguishing between PTSD with and without the dissociative subtype may examine patterns of correlation between cerebellar lobule V and hyperarousal and dissociation symptom scores in order to identify more precisely the role of this region in emotion regulation in PTSD.

The role of the cerebellum has been expanded recently to reflect its modulatory influence on the maintenance of a homeostatic baseline between low-level brainstem and midbrain activation and high-level limbic and cortical processing.^100,101^ Here, the cerebellum is thought to integrate information across these levels to smooth transitions between different emotional states.^101^ Evidence for this theory arises from the low- and high-level networks that the cerebellum is involved in,^102,103^ lesion studies demonstrating emotional impairments following cerebellar damage,^53,104^ and the effect that cerebellar inhibition has on limbic dysregulation.^48^ The right lobule V showed a significant decrease in activation in our PTSD sample as compared to controls during the presentation of subliminal threat.^54^ This effect likely contributes to symptoms of emotional impairment in PTSD and is further supported by studies that report reduced cerebellar volumes in PTSD.^105,106^ Whether reduced volumes occur as a result of trauma or are a predisposing characteristic to PTSD remains to be elucidated.

### Limitations

There are several limitations to the present study. To begin, the results reported here rely on a small sample size. Replication with a larger sample size may reveal additional between-group differences in neural activation. In particular, we predict that the insignificant findings for the negatively valenced facial expressions in the PTSD group are the result of reduced power as well as the stimuli not representing learned associations to trauma unlike the trauma-related words. Moreover, an increased patient sample could allow researchers to differentiate between persons with PTSD with and without the dissociative subtype. The subtype is distinguishable in both neural and clinical characteristics, which may be reflected in differential midbrain, brainstem, and cerebellar activation.^9,94^ In addition, the control group included in the present study represents a healthy control sample as opposed to a trauma-exposed control. As such, any discrepancies in activation cannot be definitively attributed to the PTSD diagnosis, as they may arise as a product of trauma exposure and not the subsequent development of PTSD. Notably, however, trauma-exposed controls are not a perfect comparison group as early life trauma prior to PTSD onset and the type of trauma experienced are rarely controlled for in these samples.^107^ Furthermore, the present study matched trauma-related and NWs for syllable and letter length but not for frequency of occurrence in the English language. As a result, the personalized TWs may have had unanticipated effects of novelty that could promote greater activation. Finally, trauma-related words were used as our stimuli of focus due to the high limbic activation that is reported during their presentation.^5,9,108^ However, words may not be considered a “natural” source of threat. Hence, it remains unclear whether these responses reflect the detection of a current threat in the environment or rather a reminder of a past threat. Here, different interpretations of the responses may be proposed depending upon this.

As a point of caution, the authors urge readers to not conceptualize the IAS as entirely separate from supraliminal circuits of threat detection. It is only through experimental procedures that employ brief durations of presentation and backward masks that stimuli may be presented as subliminal. Generally, the IAS should be conceptualized as a “head-start” pathway that rapidly processes salient and threatening stimuli in the environment prior to the onset of more conscious systems. Here, future research is urged to study the activation of the IAS over longer durations of time to determine whether its activation reduces when conscious systems are online or whether the IAS remains an active pathway that is perpetually a few steps ahead of conscious processes.

## Conclusion

Despite these limitations, our results further highlight the involvement of the IAS in the psychopathology of PTSD. Using improved normalization methods, we demonstrated a significant increase in midbrain activation for persons with PTSD as compared to healthy controls during the subliminal presentation of threat. These midbrain structures are known to detect threat in the environment as well as to orient toward the threat and prime defensive responses. Crucially, overactivation of these systems may lead to emotional dysregulation in PTSD—as perception is biased toward perceiving threat. In turn, the cerebellum, a region thought to attenuate emotional responses, demonstrates reduced activation during the subliminal presentation of threat in PTSD as compared to controls. In summary, this heightened inclination to perceive the world through a threatening lens coupled with a reduced ability to regulate threat-detection circuitry may have profound implications for treatment of PTSD.
